# Impact of Enhanced Recovery After Surgery with Neuromuscular Monitoring and Sugammadex on Healthcare Costs and Effectiveness of Recovery in Patients Following Anterior Cervical Spine Discectomy

**DOI:** 10.3390/jpm15030087

**Published:** 2025-02-26

**Authors:** Hung-Te Hsu, Szu-Yu Chen, Yu-Kai Huang, Kuang-I Cheng, Shih-Feng Weng, Zhi-Fu Wu

**Affiliations:** 1 Department of Anesthesiology, Kaohsiung Medical University Hospital, Kaohsiung Medical University, Kaohsiung 807, Taiwan; hdhsu@kmu.edu.tw (H.-T.H.); 1060557@mail.kmuh.org.tw (S.-Y.C.); kuaich@kmu.edu.tw (K.-I.C.); 2 Department of Anesthesiology, Faculty of Medicine, College of Medicine, Kaohsiung Medical University, Kaohsiung 807, Taiwan; 3 Department of Surgery, Division of Neurosurgery, Kaohsiung Medical University Hospital, Kaohsiung Medical University, Kaohsiung 807, Taiwan; 1000452@ms.kmuh.org.tw; 4 Department of Healthcare Administration and Medical Informatics, Kaohsiung Medical University, Kaohsiung 807, Taiwan; sfweng@kmu.edu.tw; 5 Department of Anesthesiology, Tri-Service General Hospital and National Defense Medical Center, Taipei 114, Taiwan; 6 Center for Regional Anesthesia and Pain Medicine, Wan Fang Hospital, Taipei Medical University, Taipei 116, Taiwan

**Keywords:** anterior cervical spine surgery, enhanced recovery after surgery, postoperative period, length of stay, economy, neuromuscular monitoring, sugammadex

## Abstract

**Background/Objectives**: Anterior cervical spine surgery (ACSS) is an effective surgical procedure used to treat degenerative cervical spine disease. Enhanced recovery after surgery (ERAS) is a new and promising paradigm for ACSS. The purpose of this study is to investigate the role of neuromuscular monitoring with sugammadex in the ERAS protocol, which had not been confirmed in ACSS. **Methods**: In this retrospective study, the electronic medical records of patients aged 20 to 80 years who had undergone first-time ACSS performed in the period from 1 December 2018 to 31 December 2023 were reviewed. Patients were divided into ERAS and non-ERAS groups. Inverse probability of treatment weighting (IPTW) was used to balance differences between the groups. Statistical analyses were conducted using SPSS 20, including independent samples t-tests, chi-square tests, linear regression, and logistic regression. **Results**: A total of 394 patients were included in this study: 163 in the non-ERAS group and 231 in the ERAS group. In the ERAS group, significant reductions were observed in several key outcomes compared with the non-ERAS group: LOS was reduced by 0.62 days (*p* < 0.001), hospital costs were lowered by NTD 13,174.40 (*p* < 0.001), ventilator time was decreased by 149.40 min (*p* < 0.001), time to first oral intake was shortened by 4.71 h (*p* < 0.001), and time to first ambulation was reduced by 8.00 h (*p* < 0.001). No significant differences in complication rates were observed between the two groups. **Conclusions**: The ACSS-tailored ERAS pathway with NMM and sugammadex can reduce LOS, cost, and speed of patient recovery without increasing complications.

## 1. Introduction

Anterior cervical spine surgery (ACSS) is a common surgical procedure designed to relieve spinal cord or nerve root pressure due to herniated discs or bone spurs. This procedure, while effective, can involve significant postoperative pain, respiratory complications, and extended recovery periods, impacting both patient quality of life and healthcare costs. However, this procedure has its own risks and complications. A long list of complications may arise after ACSS, including dysphagia, retropharyngeal edema, and postoperative hematoma [1]. The incidence of airway obstruction has also been reported to be as high as 6.1% after cervical spine surgery [2,3,4,5].

Although airway obstruction is uncommon after ACSS, it is a potentially life-threatening event. A study found that patients who developed respiratory compromise had high rates of 30-day mortality and morbidity, with unplanned reoperations and pneumonia being the most common complications [6]. Therefore, several studies have utilized delayed extubation protocols following ACSS based on patient risk factors and found reduced postoperative airway complications and reintubation rates [1]. However, delayed extubation was associated with longer ICU and hospital stays in ACSS patients [7]. Therefore, surgeons and anesthesiologists should communicate and understand the related risk factors before deciding on immediate or delayed extubation.

Enhanced recovery after surgery (ERAS) is an evidence-based care improvement process for surgical patients. Implementation of ERAS programs results in major improvements in clinical outcomes and costs, making ERAS an important example of value-based care applied to surgery [8,9]. Many aspects of ERAS protocols with early extubation have been introduced for ACSS [10,11,12,13,14]. However, in a cohort analysis from the Canadian Spine Outcomes and Research Network, of the eight institutions (57.1%) with an ERAS protocol, only three reported using an ERAS protocol specific to patients undergoing degenerative cervical spine surgery [15]. This is because there are concerns about whether to extubate patients immediately after ACSS, as residual muscle relaxants could cause breathing difficulties necessitating immediate reintubation. In our institution, neurosurgeons and anesthesiologists have adhered to the ERAS protocol for performing surgery and administering anesthesia for years. Recently, we integrated neuromuscular monitoring (NMM) and the use of sugammadex into the ERAS protocol for many types of surgery, including ACSS.

NMM involves the use of devices to measure the depth of neuromuscular blockade during surgery, allowing for precise control of muscle relaxation. In ACCS, this precision is critical as it facilitates better surgical access and reduces the risk of intraoperative complications. In addition, ACSS has the potential for postoperative respiratory complications due to residual neuromuscular blockade. NMM helps to mitigate this risk by providing real-time feedback on the patient’s neuromuscular function, allowing for appropriate dosing and timely reversal of muscle relaxants.

Sugammadex is a selective relaxant binding agent that provides rapid and complete reversal of rocuronium. Unlike traditional reversal agents, sugammadex works by encapsulating the neuromuscular blocking agent, thereby preventing it from binding to its receptors, which can help reduce the risk of residual paralysis and associated complications such as upper airway obstruction, reintubation, atelectasis, pneumonia, and longer hospital stays.

The ease of administering anesthesia in an ERAS protocol is not described for NMM and sugammadex in other hospitals’ protocols. Therefore, we are interested in establishing and implementing an ERAS pathway with NMM and sugammadex for ACSS to explore the economic benefits and early recovery advantages associated with these innovations in patients following ACSS.

## 2. Materials and Methods

### 2.1. Data Resources

**Figure 1:**
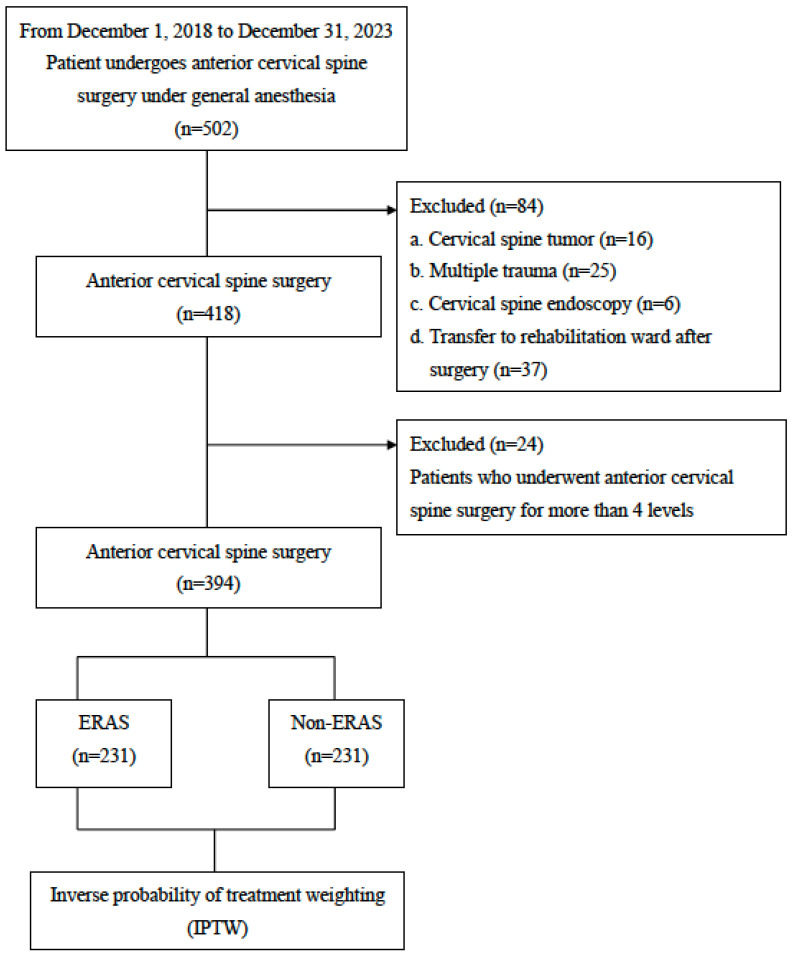
Flow diagram showing study selection process.

This retrospective study was conducted at the Kaohsiung Medical University Hospital (KMUH), Kaohsiung, Taiwan, and approved by the Institutional Review Board of the KMUH (KMUHIRB-E(II)-20240268). This study analyzed the electronic medical records from the KMUH for all patients aged 20 to 80 years who underwent general anesthesia for first-time ACSS due to cervical radiculopathy and/or myelopathy caused by degenerative disc disease and/or cervical spinal canal stenosis over a 5-year period (1 December 2018 to 31 December 2023). The study population was divided into two groups: those managed with an ERAS protocol and those without (Figure 1).

Patients with cervical spine tumors, multiple trauma injuries, those undergoing private cervical spine endoscopic surgery, four-level anterior cervical spine surgery, posterior cervical spine surgery, or combined anterior and posterior cervical spine surgery, and an American Society of Anesthesiologists (ASA) physical status classification of ≥4 were excluded due to the increased complexity and potential for higher complication rates associated with these conditions and procedures, which could confound this study’s outcomes.

### 2.2. Anesthesia

All patients fasted overnight before the procedure, and no medications were administered before the induction of anesthesia. Each patient received standard monitoring, including electrocardiography (lead II), noninvasive blood pressure, pulse oximetry, end-tidal carbon dioxide, and direct arterial blood pressure monitoring. In addition, all patients underwent monitoring for the bispectral index (BIS, BIS™ Complete 2-Channel Monitor, COVIDIEN, Boulder, CO, USA). Participants were preoxygenated with 6 L/min 100% oxygen via a facial mask to achieve peripheral oxygen saturation of 99–100% before induction.

Patients were induced with 2% lidocaine 1.0 mg/kg, an effect site concentration of 2.0–4.0 ng/mL of remifentanil (50 mcg/mL, Minto model), and an effect site concentration of 3.0–6.0 mcg/mL of propofol (10 mg/mL; Schnider model), with continuous infusion using two separate TCI pumps or 1.0–2.0 mcg/kg of intravenous fentanyl and 1.0–2.0 mg/kg of propofol. Rocuronium (0.6 mg/kg) was administered after loss of consciousness in all patients to facilitate endotracheal intubation. Next, 5 mg of dexamethasone was prescribed before and after intubation to prevent postoperative nausea and vomiting. Inhalational anesthesia was maintained with a total fresh gas flow of 1.0–1.5 L/min. A lung-protective strategy was prescribed, including FiO_2_ < 40%_,_ low tidal volume (<6–8 mL/kg of predicted body weight), plateau (<28–30 cmH_2_O), and driving pressure (<15 cmH_2_O). In addition, end-tidal carbon dioxide was maintained at 35–45 mmHg by adjusting the ventilation rate. The intraoperative administration of propofol or sevoflurane or desflurane combined with opioids was guided by maintaining the BIS value at 40–60 and hemodynamics within the baseline of ±20% during surgery. In addition, continuous infusion of dexmedetomidine was applied at a rate of 0.1–0.2 mcg/kg/h in some patients of both groups. To maintain moderate to deep neuromuscular blockade as guided by the train-of-four (TOF) count (NMT Mechano Sensor, GE Healthcare, Chicago, IL, USA), we checked the TOF of each patient every 15 min during the operation to keep the TOF count at between 0 and 1 to prevent sudden patient movement during surgery. In the ERAS group, while the operation was completed, patients who had received a dose of sugammadex of 2.0 mg/kg before tracheal extubation and who were extubated at TOF > 0.9 were then transferred to the post-anesthesia care unit or surgical intensive care unit (SICU) after surgery. In contrast, patients in the non-ERAS group without extubation were transferred to the SICU for further care. In the SICU, the time of extubation and time to oral intake were determined by the attendant physicians, and time to oral intake and ambulation were determined by the surgeon’s order on the ward. Postoperative pain management was administered with 40 mg intravenous parecoxib sodium, 1.0 g propacetamol, 1.0 mg/kg tramadol, or opioids as needed.

### 2.3. Outcomes

The primary outcomes were length of stay (LOS) and hospital costs (New Taiwan dollars, NTD). Secondary outcomes included time to first oral intake, time to first ambulation, complications, ICU ventilator time, ICU admission rate, and 90-day readmission rate.

### 2.4. Statistical Analysis

Differences in demographic data were analyzed using independent samples t-tests for continuous variables and the chi-square tests for categorical variables. To address the imbalance between the ERAS and non-ERAS groups regarding demographic characteristics (age, sex, body mass index (BMI), and smoking), ASA, comorbid factors (hypertension, diabetes mellitus (DM), and coronary artery disease (CAD)), and clinical features (surgery level, anesthesia technique, postoperative analgesics, anesthesia time, and surgical time), the inverse probability of treatment weighting (IPTW) method was employed. After that, linear and logistic regression analyses were conducted to assess relationships between various outcomes, including ICU ventilator time, ICU admission rate, LOS, hospital costs, time to first oral intake, time to ambulation, and complications such as dysphagia, postoperative nausea and vomiting, Horner syndrome, hematoma, reoperation, reintubation, surgical site infection, dyspnea, cardiovascular complications, pulmonary complications, and 90-day readmission rate. Statistical significance was set at *p* < 0.05. All analyses were performed using SPSS Statistics Version 20.0 (IBM Corp., Armonk, NY, USA).

## 3. Results

### 3.1. Patient Characteristics

A total of 394 patients were included in this study, with 163 in the non-ERAS group and 231 in the ERAS group (Figure 1). Demographic data and comorbidities are detailed in Table 1. Before applying IPTW, significant differences were observed between the groups: the ERAS group was younger than the non-ERAS group (54.72 ± 11.63 vs. 57.51 ± 11.02 years, *p* = 0.014), and a higher proportion of patients in the non-ERAS group had diabetes compared with the ERAS group (22.1% vs. 13.0%, *p* = 0.025). Additionally, the distribution of surgical levels differed significantly between groups, with a higher proportion of one-level ACSS in the ERAS group and three-level ACSS in the non-ERAS group (*p* < 0.001). Furthermore, the ERAS group received more intravenous anesthesia compared with the non-ERAS group (33.3% vs. 15.3%, *p* < 0.001) and more postoperative analgesics (47.2% vs. 14.1%, *p* < 0.001). The anesthesia time (218.53 ± 59.36 vs. 265.62 ± 71.26 min, *p* < 0.001) and surgical time (166.18 ± 57.82 vs. 214.34 ± 67.27 min, *p* < 0.001) were significantly shorter in the ERAS group compared with the non-ERAS group. However, after adjusting for these variables using IPTW, the differences between the groups became non-significant, including age (56.58 ± 11.13 vs. 56.39 ± 11.48 years, *p* = 0.8), diabetes prevalence (20.5% vs. 18.6%, *p* = 0.6), surgical level distribution (*p* = 0.4), intravenous anesthesia (22.3% vs. 18.9%, *p* = 0.3), postoperative analgesia (29.9% vs. 27.5%, *p* = 0.5), anesthesia time (251.10 ± 75.86 vs. 244.01 ± 68.24 min, *p* = 0.2), and surgical time (198.56 ± 73.74 vs. 193.03 ± 63.49 min, *p* = 0.3).

### 3.2. Descriptive Analysis Between ERAS Group and Non-ERAS Group After IPTW

A descriptive analysis between the ERAS group and non-ERAS group after IPTW is shown in Table 2. LOS was significantly shorter in the ERAS group compared with the non-ERAS group, with mean values of 5.69 days and 6.32 days, respectively (*p* < 0.001). Hospital costs were also significantly lower in the ERAS group (NTD 134,436.66 ± 45,005.77) compared with the non-ERAS group (NTD 147,070.22 ± 60,946.94), with a *p*-value of 0.001. Additionally, the time to first oral intake was significantly reduced in the ERAS group (8.02 ± 3.10 h) compared with the non-ERAS group (12.91 ± 4.72 h, *p* < 0.001). The time to first ambulation was also significantly shorter in the ERAS group (18.19 ± 8.70 h) compared with the non-ERAS group (25.95 ± 12.11 h, *p* < 0.001). The ICU ventilator time (0 vs. 161.06 ± 200.91 min, *p* < 0.001), ICU admission rate (28.9% vs. 100%, *p* < 0.001), and ICU stay (0 vs. 1 days, *p* < 0.001) were significantly lower in the ERAS group than the non-ERAS group.

The analysis also assessed the incidence of complications. No significant differences in complication rates were observed between the ERAS and non-ERAS groups (Table 2). There were two cases of neck hematoma in the ERAS group; one was managed conservatively, while the other required reoperation. In the non-ERAS group, there was one case of neck hematoma that required reintubation and reoperation. Additionally, two patients in the non-ERAS group experienced laryngeal edema with prolonged mechanical ventilation for 2 days, which was treated with steroids. There was no postoperative nausea and vomiting, Horner syndrome, surgical site infection, dyspnea, cardiovascular complications, or pneumonia. Furthermore, there were no 90-day readmissions in either group. Statistical analysis indicated that participation in the ERAS program did not increase the likelihood of these complications.

### 3.3. Outcomes of Linear Regression Analysis Comparing ERAS and Non-ERAS Groups

The outcomes of the linear regression analysis comparing ERAS and non-ERAS groups are presented in Table 3. The adjusted model controlled for age, sex, BMI, smoking, ASA, hypertension, DM, CAD, surgical level, anesthesia technique, anesthesia time, surgical time, and postoperative analgesics. The multivariate analysis of LOS revealed a significant reduction of 0.62 days in the ERAS group compared with the non-ERAS group (*p* < 0.001). Similarly, hospital costs were significantly lower in the ERAS group, with a reduction of NTD 13,174.40 compared with the non-ERAS group (*p* < 0.001). The analysis of ventilator time showed a significant reduction of 149.40 min in the ERAS group compared with the non-ERAS group (*p* < 0.001), and analysis of the time to first oral intake showed a significant reduction of 4.71 h in the ERAS group compared with the non-ERAS group (*p* < 0.001). Additionally, the time to first ambulation was reduced by 8.00 h in the ERAS group compared with the non-ERAS group (*p* < 0.001, Table 3).

## 4. Discussion

The major finding of this study is that implementation of an ERAS protocol for ACSS in our hospital significantly decreased LOS and cost and improved patient recovery without increasing 90-day readmission rates, pulmonary complications, and reoperation rates. The use of NMM and sugammadex in ACSS significantly enhances patient safety by minimizing the risk of residual neuromuscular blockade, and rapidly reversing neuromuscular blockade facilitates earlier extubation, mobilization, oral intake, and recovery. This results in swift recovery of muscle function, reducing the time patients spend in the ICU and hospital. In our hospital, the surgeon and anesthesiologists collaborate to determine the timing of extubation. Most patients in the ERAS group were transferred to a general ward. In total, 28.9% of patients were transferred to the SICU for observation after extubation as we had concerns about airway complications.

A retrospective cohort study by Soffin et al. [12] analyzed 33 patients who underwent ACSS with an ERAS protocol, showing minimal complications and no readmissions within 90 days, though there were isolated cases of dyspnea, hypoxia, and dysphagia. Debono et al. [16] compared outcomes after ACSS before (n = 749) and after (n = 612) ERAS pathway implementation. The introduction of the ERAS approach was associated with decreased LOS (3.1 vs. 1.3 days). Furthermore, Debono et al. [10] conducted a propensity score matching (PSM) study and found that LOS was reduced by 1.6 days in ERAS patients. In addition, Wang et al. [11] reported an ERAS protocol for ACSS that included 135 patients in the ERAS group and 122 patients in the non-ERAS group. There was a significant decrease in LOS in the ERAS group (8.7 vs. 10.4 days) in patients aged over 60 years who received ACSS. However, total major complications affected 5.2% of patients in the ERAS group. Recently, in a quantitative meta-analysis including 10 articles, Qin et al. [17] reported that the implementation of an ERAS protocol decreases LOS, cost, and complication rates, and improves satisfaction for patients undergoing ACSS. The results of our study aligned with these findings, showing reduced LOS, lower hospital costs, and no increase in complications. Mesfin et al. [18] reported the establishment and implementation of an ERAS for ACSS at same-day discharge. The 30-day readmission rates (%) were 2.7, 3.9, 4.5, and 15 in one-, two-, three-, and four-level ACSS, respectively. Accordingly, hospitalization for several days following ACSS is still the rule in Taiwan, as the safety of patients before discharge must be ensured. Therefore, an ambulatory procedure or out-patient procedure is not applicable to ACSS patients in Taiwan.

ACSS is a highly standardized procedure known for its efficiency and low mortality rate. However, there are rare but potentially catastrophic complications that can occur during the immediate perioperative period, with respiratory compromise being a significant concern. Airway complications have been reported at rates from 0.1% to 5.2% [1,5,6,7,19,20,21,22]. In our study, the incidence of neck hematoma was 0.87% (2/231) and reintubation 0.44% (1/231) in the ERAS group, which is comparable to previous studies. In a multicenter retrospective cohort study of North America, Nagoshi et al. [21] reported that 9 out of 8887 patients (0.1%) required reintubation and rates ranged from 0% to 0.59% across participating institutions. The time to the development of airway symptoms after surgery was within 24 h. Consequently, many surgeons opt for delayed extubation after surgery to reduce the risk of fatal complications, often recommending at least one night of postoperative monitoring in SICU. However, Raksakietisak et al. [23] analyzed 506 patients and found that delayed extubation occurred in 116 patients (22.9%), with 15 (3.0%) requiring reintubation. Furthermore, Jing et al. [7] reported that delayed extubation was associated with longer ICU and hospital stays in ACSS patients because early recognition of these postoperative hemorrhages, and appropriate management, are critical for optimizing recovery and limiting morbidity and mortality after ASCC. Thus, early extubation is considered a preferable approach, and anesthesiologists should carefully assess the related risk factors before deciding on immediate or delayed extubation. Therefore, we recommend the perioperative use of NMM combined with sugammadex for ACSS, in line with practice guidelines from the European Society of Anaesthesiology and Intensive Care and the American Society of Anesthesiologists [24,25] after ACSS.

Recently, Carr et al. [26] reported that perioperative use of NMM was associated with reduced odds of postoperative pulmonary complications (PPCs) and 90-day mortality. Cheng et al. [27] demonstrated that patients who received NMM with sugammadex had a lower risk of PPCs compared with those who received neostigmine plus glycopyrrolate after da Vinci surgery for malignancy. Similarly, Min et al. [28] reported that based on using a generalized linear model in a PSM cohort, sugammadex use was associated with a 6% decrease in LOS as compared with neostigmine use in robot-assisted laparoscopic prostatectomy. Furthermore, Bardia et al. [29] conducted a randomized trial that showed that the administration of sugammadex after cardiac surgery decreased time to extubation by approximately 1 h. Song et al. [30] reported that the median LOS was shorter (10.0 vs. 12.0 days) and the incidence of PPCs was lower (18.1 vs. 29.9%) in a sugammadex group after open lobectomy for lung cancer. Recently, Olesnicky et al. [31] conducted a systematic review and meta-analysis involving 11 trials and examined the effect of sugammadex on days alive and out of the hospital for up to 30 days and other important patient outcomes. The observed difference in mortality (odds ratio 0.39 [0.15–1.01]) is considered clinically significant and warrants further investigation. Moreover, sugammadex is associated with a reduction in PPCs; however, this might not translate to a difference in LOS, quality of recovery, or mortality. In addition, in a PSM analysis after open lumbar spine surgery, the utilization of sugammadex did not lead to a reduction in LOS but was associated with a decreased incidence of PPCs [32]. Furthermore, sugammadex reversal significantly hastened neuromuscular blockade recovery compared with neostigmine reversal in geriatric patients after open lumbar spine surgery. It significantly decreased operating room time but not PACU time or LOS [33]. While the role of NMM and sugammadex in reducing PPCs has been demonstrated, further investigation is needed to determine its impact on LOS or outcomes across different types of surgery. Accordingly, to date, there have been very few studies on NMM with sugammadex in the ERAS protocol of ACSS. Our results suggest that the integration of NMM and sugammadex during ACSS improves intraoperative efficiency and enhances postoperative recovery. As NMM ensures optimal muscle relaxation and precise control during surgery, it reduces surgical time and improves surgical outcomes. In addition, sugammadex enables the rapid and complete reversal of neuromuscular blockade, allowing for quicker patient recovery from anesthesia and reducing the need for prolonged postoperative ICU monitoring. These efficiencies lead to better utilization of ICU beds, ultimately lowering overall healthcare costs.

Our study has several limitations. Firstly, being a retrospective, single-center observational study, our findings may not be generalizable to other hospitals, necessitating further investigation. Secondly, the sample size was small, with patients not randomly allocated and the two groups unequal in size. This imbalance occurred due to the growing acceptance of ERAS by both surgeons and patients; there is a trend toward an increasing number of patients. On the other hand, some surgeons did not participate in the ERAS protocol, and patients who did not wish to self-pay NTD 6000 for NMM and sugammadex were placed in the non-ERAS group, resulting in a larger ERAS group. In epidemiology, it is important for the control group to be comparable to the study group in certain characteristics to avoid confounding variables. To address this, we used the IPTW method, which calculates propensity scores using logistic regression, assigning weights to cases that may be greater or less than 1. The advantage of IPTW is that it allows for the analysis of the entire dataset, making the results more generalizable to the population than PSM and providing better external validity. Therefore, we chose IPTW over PSM for this study.

## 5. Conclusions

This study shows that the integration of neuromuscular monitoring and sugammadex into ERAS protocols for anterior cervical spine surgery offers substantial economic benefits and promotes early recovery. These advancements not only reduce healthcare costs through shorter hospital stays and efficient ICU resource utilization but also improve patient outcomes by facilitating a quicker return to daily activities. As healthcare systems continue to seek ways to optimize care delivery and reduce costs, the adoption of these innovations represents a promising step forward in anterior cervical spine surgery.

## Figures and Tables

**Table 1:** Baseline characteristics between ERAS group and non-ERAS group.

Variable		Before IPTW			After IPTW		
		ERAS (N = 231) Mean ± SD N(%)	Non-ERAS (N = 163) Mean ± SD N(%)	*p*-Value	ERAS Mean ± SD (%)	Non-ERAS Mean ± SD (%)	*p*-Value
Demographic characteristics							
Age		54.72 ± 11.63	57.51 ± 11.02	0.014	56.58 ± 11.13	56.39 ± 11.48	0.8
Sex	male	113 (48.9)	85 (52.1)	0.6	(53.2)	(50.4)	0.5
	female	118 (51.1)	78 (47.9)		(46.8)	(49.6)	
BMI		25.24 ± 3.91	25.63 ± 4.35	0.4	26.00 ± 4.23	25.67 ± 4.11	0.3
Smoking		30(13.0)	33 (20.2)	0.072	(20.8)	(17.8)	0.3
ASA	I and II	120 (51.9)	69 (42.3)	0.075	(51.2)	(47.0)	0.3
	III	111 (48.1)	94 (57.7)		(48.8)	(53.0)	
Comorbid factors							
Hypertension		56(24.2)	55 (33.7)	0.5	(36.6)	(30.5)	0.084
Diabetes		30(13.0)	36 (22.1)	0.025	(20.5)	(18.6)	0.6
CAD		6(2.6)	4 (2.5)	1.0	(2.0)	(2.0)	1.0
Clinical characteristics							
Level	I	103 (44.6)	27 (16.6)	<0.001	(28.5)	(31.1)	0.4
	II	94 (40.7)	60 (36.8)		(34.4)	(36.0)	
	III	34 (14.7)	76 (46.6)		(37.1)	(32.9)	
Anesthesia method	intravenous	77 (33.3)	25 (15.3)	<0.001	(22.3)	(18.9)	0.3
	inhalational	154 (66.7)	138 (84.7)		(77.7)	(81.1)	
Postoperative analgesics	0–1	122 (52.8)	140 (85.9)	<0.001	(70.1)	(72.5)	0.5
	≥2	109 (47.2)	23 (14.1)		(29.9)	(27.5)	
Anesthesia time (min)		218.53 ± 59.36	265.62 ± 71.26	<0.001	251.10 ± 75.86	244.01 ± 68.24	0.2
Surgical time (min)		166.18 ± 57.82	214.34 ± 67.27	<0.001	198.56 ± 73.74	193.03 ± 63.49	0.3

IPTW—inverse probability of treatment weighting; ERAS—enhanced recovery after surgery; BMI—body mass index; ASA—American Society of Anesthesiologists physical status classification; CAD—coronary artery disease; LOS—length of stay; mean ± standard deviation/N(%).

**Table 2:** Descriptive analysis between ERAS group and non-ERAS group after IPTW.

Variable	After IPTW		
	ERAS Mean ± SD (%)	Non-ERAS Mean ± SD (%)	*p*-Value
Medical expenses			
LOS	5.69 ± 1.86	6.32 ± 3.06	<0.001
Cost (NTD)	134,436.66 ± 45,005.77	147,070.22 ± 60,946.94	0.001
Ventilator time (minutes)	0	161.06 ± 200.91	<0.001
ICU admission	(28.9)	(100)	<0.001
Complications			
Neck hematoma	(0.7)	(0.6)	1.000
Reoperation	(0.2)	(0.6)	0.8
Pulmonary complications	(0)	(1.1)	0.080
Postoperative recovery indicators			
First oral intake time (hours)	8.02 ± 3.10	12.91 ± 4.72	<0.001
First ambulation time (hours)	18.19 ± 8.70	25.95 ± 12.11	<0.001

IPTW—inverse probability of treatment weighting; mean ± standard deviation/N (%); ERAS—enhanced recovery after surgery; LOC—length of stay; NTD—New Taiwan dollars.

**Table 3:** Outcomes of linear regression analysis comparing ERAS and non-ERAS groups.

Variable	Unadjusted Model		*p*-Value	Adjusted Model		*p*-Value
	Beta	95%CI		Beta	95%CI	
LOS						
ERAS vs. non-ERAS	−0.63	−0.98~−0.29	<0.001	−0.62	−0.94~−0.29	<0.001
Cost (NTD)						
ERAS vs. non-ERAS	−12,633.60	−20,032.01~−5235.10	0.001	−13,174.40	−20,306.10~−6042.60	<0.001
Ventilator time (min)						
ERAS vs. non-ERAS	−161.56	−179.80~−142.30	<0.001	−149.40	−166.30~−132.60	<0.001
First oral intake time (hrs)						
ERAS vs. non-ERAS	−4.89	−5.44~−4.34	<0.001	−4.71	−5.22~−4.20	<0.001
First ambulation time (hrs)						
ERAS vs. non-ERAS	−7.76	−9.21~−6.30	<0.001	−8.00	−9.38~−6.62	<0.001

Adjusted model was adjusted according to age, sex, body mass index, smoking, American Society of Anesthesiologists physical status classification, hypertension, diabetes, coronary artery disease, surgical level, anesthesia technique, anesthesia time, surgical time, and postoperative analgesics. LOS—length of stay; ERAS—enhanced recovery after surgery; NTD—New Taiwan dollars.

## Data Availability

The anonymized data processed for this study can be made available to researchers upon reasonable request.
